# Prognostic indicators in hepatocellular carcinoma: a systematic review of 72 studies

**DOI:** 10.1111/j.1478-3231.2008.01957.x

**Published:** 2009-04

**Authors:** Puneeta Tandon, Guadalupe Garcia-Tsao

**Affiliations:** 1 Digestive Diseases Section, Yale University School of MedicineNew Haven, CT, USA; 2 VA Connecticut Healthcare SystemWest Haven, CT, USA; 3 Division of Gastroenterology, University of AlbertaEdmonton, AB, Canada

**Keywords:** hepatocellular carcinoma, multivariable analysis, prognosis

## Abstract

**Background:**

Although there are many studies of the predictors of death in hepatocellular carcinoma (HCC), most combine patients with and without cirrhosis and many combine those with compensated and decompensated cirrhosis.

**Objective:**

To perform a systematic review of the literature evaluating the predictors of death in patients with cirrhosis and HCC and to evaluate whether the predictors differ between patients with compensated and decompensated cirrhosis.

**Methods:**

Inclusion criteria: (i) publication in English, (ii) adult patients, (c) >80% of the patients had cirrhosis, (iv) follow-up >6 months and (v) multivariable analysis. Quality was based on the accepted quality criteria for prognostic studies.

**Results:**

Of the 1106 references obtained, 947 were excluded because they did not meet the inclusion criteria. A total of 23 968 patients were included in 72 studies (median, 177/study); 77% male, median age 64, 55% Child–Pugh class A. The most robust predictors of death were portal vein thrombosis, tumour size, α-foetoprotein and Child–Pugh class. Sensitivity analysis using only 15 ‘good’ studies and 22 studies in which all patients had cirrhosis yielded the same variables. In the studies including mostly compensated or decompensated patients, the predictors were both liver and tumour related. However, these studies were few and the results were not robust.

**Conclusions:**

This systematic review of 72 studies shows that the most robust predictors of death in patients with cirrhosis and HCC are tumour related and liver related. Future prognostic studies should include these predictors and should be performed in specific patient populations to determine whether specific prognostic indicators are more relevant at different stages of cirrhosis.

Hepatocellular carcinoma (HCC) is responsible for significant morbidity and mortality in cirrhosis. It commonly leads to decompensation of cirrhosis and is one of the leading causes of death in cirrhotic patients (1, 2). Identifying the accurate prognostic indicators of death for HCC allows the provider to counsel individual patients and also forms the basis of any decision-making process. Most cases of HCC in the western world occur in the setting of cirrhosis and, therefore, prognosis is determined not only by factors related to the tumour but also by factors related to cirrhosis. In fact, current prognostic models for HCC include parameters of liver dysfunction and parameters related to HCC (3–5). However, studies on the prediction of death in HCC include patients both with and without underlying cirrhosis, and this heterogeneity may impact the clinical utility of current prognostic models.

Additionally, one could hypothesize that, as in cirrhosis not associated with HCC (2), the prognostic factors for HCC would be different in patients with underlying compensated vs. decompensated cirrhosis, with factors related to the tumour having a greater prognostic significance in the former and factors related to both tumour and liver disease being more important in the latter. The purpose of this systematic review is to evaluate the predictors of death in patients with HCC and underlying cirrhosis to determine whether the predictors differ between patients with compensated and decompensated cirrhosis.

## Methods

A Medline search was performed using the terms (survival [ALL] OR mortality [ALL] OR predictor [ALL] OR prognosis [ALL] OR prognostic [ALL] AND (multivariate OR Cox OR Cox's OR adjusted OR adjustment OR logistic [ALL]) [MESH] AND hepatocellular carcinoma [MESH]. The search was carried out in March 2008 and no lower date limit was set on the search results. Additional studies were located by manual search using references from retrieved articles.

Studies were selected using the following inclusion criteria: (i) English language, (ii) inclusion of only adult patients, (iii) established diagnosis of HCC, (iv) >80% of patients included had cirrhosis – if cirrhosis was not specifically mentioned, it was assumed that the population was comprised of cirrhotic patients if all patients in the study were classified into Child–Pugh classes, (v) survival analysis was reported, (vi) multivariate analysis of prognostic indicators of mortality was performed and (vii) patients in the study were either untreated or the study included more than one type of therapy following a local treatment protocol. Trials were excluded if they had (i) <80% cirrhosis, (ii) only prediction of short-term survival (<6 months), (iii) a single treatment option was being analysed, (iv) randomized trials comparing two different therapies, (v) the main predictive factor being studied was a tumour histological feature (i.e. tumour staining) and (vi) abstract only or full-text article not available. The rationale for excluding trials with only a single treatment option was that as candidates for particular treatment options may have varying prognoses, this may have resulted in a biased inclusion of patients. Required clinical data and data regarding study validity were predefined and collected for all studies meeting the inclusion criteria.

Study validity was assessed using predefined quality criteria, as listed in Table 1 (2, 6–8). Because none of the trials met all the quality criteria (Table 1), ‘good quality’ studies were arbitrarily defined as those that fulfilled all four of the following major quality criteria: (i) enrollment of consecutive patients, (ii) listing relevant baseline data, (iii) reporting the number of deaths and (iv) the absence of overfitting (ratio of the number of deaths/the number of variables >10). All ‘good quality’ studies documented the number of patients with cirrhosis.

**Table 1:** Summary of the characteristics of 72 studies of the predictors of mortality in hepatocellular carcinoma

Characteristics of studies	*n* (%)
Aim	
Explanatory	22 (31)
Predictive	50 (69)
Design	
Prospective	21 (29)
Retrospective	51 (71)
Inception cohort	2 (3)
Patients were included consecutively*	41 (57)
Inclusion and exclusion criteria defined	34 (47)
Number of excluded patients specified	23 (32)
Diagnosis of hepatocellular carcinoma well defined	59 (82)
Candidate variables identified *a priori*	38 (53)
Candidate variables included previously identified the predictors of survival	72 (97)
Relevant baseline data shown*†	43 (60)
Length of follow-up reported	21 (29)
Patients lost to follow-up reported	13 (18)
Number of deaths reported*	43 (60)
Causes of death reported	29 (40)
Ratio of number of deaths/number of variables >10 (i.e. no overfitting)*	28 (39)
Missing data reported	47 (65)
Results validated internally or externally	7 (10)
Geographical origin of the study	
Japan	28 (39)
Italy	19 (26)
Thailand	5 (7)
Spain	3 (4)
Sites publishing ≤2 studies‡	17 (24)

* ‘Good’ study defined by the presence of these quality variables.

† At a minimum, studies should have reported age, sex, presence and aetiology of cirrhosis and Child–Pugh class or components.

‡ Other sites included Australia, Taiwan, Hong Kong, Germany, Portugal, Kuwait, USA, France, Austria, Turkey, Greece, Belgium, Singapore and China.

An ‘advanced’ tumour was arbitrarily defined as any of the following: ‘multinodular’ tumours or tumours exceeding the Milan criteria (one tumour >5 cm or three tumors, with any of them being >3 cm) (9). In the analysis aimed at determining whether the prognostic variables differed between patients with advanced vs. non-advanced tumours, studies including mostly advanced tumours were defined as those in which 66% of the patients had advanced tumours and studies including mostly non-advanced tumours were defined as those in which >66% of the patients had non-advanced tumours. Decompensated cirrhosis was defined as a patient with Child–Pugh B or C classification. In the analysis aimed at determining whether the prognostic variables differed between patients with compensated vs. decompensated cirrhosis, studies including mostly compensated patients were arbitrarily defined as those in which >66% of the patients were Child–Pugh A and studies including mostly decompensated patients were arbitrarily defined as those in which >66% of the patients were Child–Pugh B or C.

The 1-, 2-, 3- and 5-year cumulative survivals and the final cumulative survival were recorded if present. The first five significant prognostic indicators in each multivariate analysis were recorded as were the number of studies evaluating each variable. A sensitivity analysis was performed using the ‘good quality’ studies and using the studies in which 100% of the patients had cirrhosis.

## Results

**Fig. 1:**
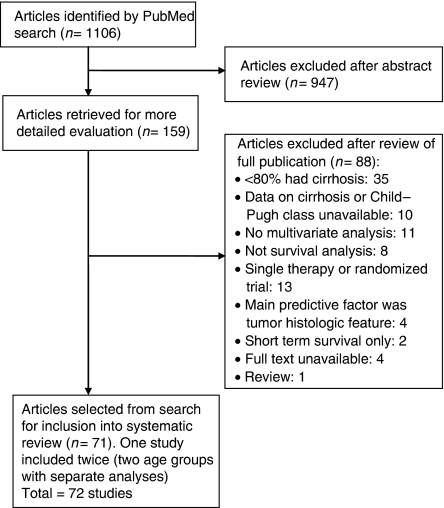
Trial flow.

As shown in Figure 1, of the 1106 references identified through a Medline search, 947 were excluded on analysis of the abstract provided because they did not meet the inclusion and exclusion criteria. Of the remaining 159 full-text articles, 88 were excluded, 84 because they did not meet the inclusion criteria (at least 35 had combined cirrhotic and non-cirrhotic patients, with <80% being cirrhotic and 10 did not even mention whether there was underlying cirrhosis) and four because the full-text paper was not found, leaving 71 articles (4, 10–79) meeting the criteria for inclusion in the review. One trial (46) was included twice as it had two separate analyses for two different age groups, bringing the total number of studies reviewed to 72.

### Description of the prognostic studies

Regarding the aim of the study, 69% (50/72) of the studies were predictive studies, i.e. they assessed the prognostic value of multiple variables without concern on the pathophysiology of the variable, and 31% (22/72) were explanatory, i.e. a specific variable was evaluated according to a biologically plausible hypothesis (Table 1). Regarding the design of the study, 71% were retrospective and only 29% were prospective. Only 57% of the studies enrolled patients consecutively. The least commonly fulfilled quality criteria were the presence of an inception cohort (3%), internal/external validation of results (10%) and reporting of loss to follow-up (18%). No study fulfilled all quality criteria. Fifteen studies met our definition for a ‘good quality study’, i.e. they fulfilled the four major predefined quality criteria (4, 21, 26, 31, 33, 34, 38, 44, 53, 56, 58, 66, 68, 71, 77). The geographical origin of the majority of the studies was Japan or Italy (Table 1). Eighty-two per cent of the articles outlined their diagnostic criteria for HCC. This included various combinations of compatible histology, radiology and α-foetoprotein (AFP) investigations.

### Description of patients included in the prognostic studies

Overall, 23 968 patients were included in the 72 studies. Their characteristics are reported in Table 2. The median number of patients per trial was 177, with a range from 30 to 4525. The median age was 66, with 71% males. Fifty-two of the included studies explicitly stated the number of patients with cirrhosis. Although the other 20 studies did not, they did divide all patients by the Child–Pugh classification which is, by convention, reserved for cirrhotic patients. None of these 20 studies met our predefined quality criteria.

**Table 2:** Characteristics of hepatocellular carcinoma patients included in 72 studies evaluating predicting the predictors of mortality

Variable	No. of studies with available information	Median [range] (interquartile range)
Sample size (patients included/study)	72	177 [30–4525]
Age	66	64 [51–82]
% male	71	77 [63–93]
% cirrhosis	52	97 (90–100)
Aetiology of cirrhosis	68	
Hepatitis C (%)	56	60 (38–81)
Alcohol (%)	31	16 (9–29)
Hepatitis B (%)	61	18 (11–40)
Mixed (%)	29	5 (2–9)
Other (%)	46	12 (7–18)
Child–Pugh class		
A (%)	58	55 (45–65)
B (%)	53	33 (28–40)
C (%)	52	11 (7–18)
% with decompensated cirrhosis	60	45 (35–56)
% with advanced tumour	68	52 (45–58)
% with varices	9	49 (45–58)
Treatment		
Curative* (%)	51	34 (29–49)
Palliative† (%)	48	30 (22–48)
Untreated (%)	50	26 (12–56)
Other‡ (%)	25	15 (4–50)
Follow-up period (months)	21	19 (12–28)
Mortality (%)	43	69 (57–81)
Causes of death	28	
Hepatocellular carcinoma (%)	21	59 (35–74)
Progression of liver disease (%)	23	28 (18–56)
Variceal bleeding (%)	20	9 (6–15)
Sepsis (%)	7	5 (1–8)
Median survival time (months)	36	16 (5–22)
1-year cumulative survival (%)	46	66 (49–81)
2-year cumulative survival (%)	18	49 (28–56)
3-year cumulative survival (%)	32	36 (22–46)
5-year cumulative survival (%)	24	24 (15–30)
Final cumulative survival (%)	31	21 (13–29)
No. of variables assessed (total)	36	15 (11–19)
No, of deaths	43	121 (55–248)
No. variables entered on multivariate analysis	66	7 (5–10)
No. variables independently predictive of death	72	4 (2–4)

* Liver transplantation, surgical resection or local ablative therapies (percutaneous ethanol injection, radiofrequency ablation).

† Transarterial embolization or chemoembolization, systemic or hepatic arterial chemotherapy.

‡ Other treatment modalities, treatment not mentioned or combination therapy.

### Survival in the prognostic studies

As shown in Table 2, the median follow-up time was 21 months, with a mortality rate of 69%. The median survival time was 16 months. The 1-, 3-year and final cumulative survival were 66, 36 and 24% respectively. The median survival in studies including patients with predominantly decompensated cirrhosis (*n*=3 studies with available data) (38, 54, 62) was 8 months as compared with 29 months in those including patients with predominantly compensated cirrhosis (*n*=4 studies) (39, 51, 59, 74). Twenty-eight studies reported the causes of death. The most common cause of death was recorded as being related to HCC in the majority (59%), followed by progression of liver disease (28%) (Table 2).

### Prognostic variables

A total of 79 variables were evaluated in these studies (Table 3). The five most common independent predictors of death in HCC were portal vein thrombosis, tumour size, AFP, Child–Pugh class and bilirubin (Table 4). Of these, the variables that were found to be most significant in a larger number of studies were portal vein thrombosis, tumour size and Child–Pugh class (each of them evaluated in more than 30 studies and found to be significant in >50% of them). The Cancer of the Liver Italian Program (CLIP) score was the sixth independent predictor found in 11/15 studies (24, 26, 34, 36, 45, 47, 53, 54, 58, 61, 65, 66, 72, 75, 79) evaluating the variable. Variables found to be independently predictive of survival in at least one study are shown in Table 5 and, of these, lack of therapy and performance status are remarkable because they were found to be predictive of death in over two-thirds of the 15 and eight studies in which they were evaluated. Eighteen (23%) of the 79 variables evaluated were not significantly predictive of death in any study.

**Table 5:** Variables that were found to be significant in one to 10 studies (*n*=55)

Variables significant among the first five in two to 10 studies divided by the total studies in which the variable was tested (%)		Variables significant among the first five in only one study divided by the total studies in which the variable was tested (%)		Variables significant among the first five and tested in only one study
Untreated	10/15 (67)	Viral load	1/2 (50)	Centre of diagnosis
Tumour number	8/22 (36)	ICG	1/2 (50)	Modified JIS score
Albumin	7/16 (44)	Creatinine	1/3 (33)	Lymph nodes
Performance status	6/8 (75)	JIS scale	1/3 (33)	CRP
Age	6/16 (38)	Period of surveillance	1/3 (33)	α-1 antitrypsin
Treatment modality	5/7 (71)	MELD	1/5 (20)	Tumour echo
Metastases	5/8 (63)	Hepatitis C	1/6 (17)	Tumour margin
Ascites	5/15 (33)	GGT	1/7 (14)	SUV ratio
DCP^a^	4/6 (67)	ALP	1/10 (10)	Encapsulation
Gross HCC^b^	4/9 (44)			LAK
Tumour stage	4/14 (29)			TGF-β
Okuda scale	4/15 (27)			NK
LCSGJ liver damage	3/4 (75)			HBeAg
Tumour histology	3/5 (60)			Tumour doubling time
Symptoms	3/7 (43)			p53
Alcohol	2/2 (100)			IL-8
Surgery	2/2 (100)			HGF
LCSGJ stage^c^	2/2 (100)			Combination of staging systems
AFP-L3	2/2 (100)			LINE-1
BCLC scale	2/3 (67)			
Milan criteria	2/3 (67)			
Urea	2/3 (67)			
TACE	2/3 (67)			
Mode of detection	2/6 (33)			
Varices	2/6 (33)			
PT	2/8 (25)			
Hepatitis B	2/10 (20)			

AFP-L3, lens-culinaris agglutinin-reactive fraction of α-foetoprotein; ALP, alkaline phosphatase; BCLC, Barcelona Clinic Liver Cancer; CRP, C reactive protein; DCP, des-gamma-carboxy prothrombin; GGT, ã-glutamyl transpeptidase; HBeAg, hepatitis B envelope antigen; HCC, hepatocellular carcinoma; HGF, hepatocyte growth factor; ICG, indocyanine green clearance; IL, interleukin; JIS, Japanese Integrated System; LAK, lymphokine-activated killer activity; LCSGJ, Liver Cancer Study Group of Japan; MELD, model for end-stage liver disease; NK, natural killer activity; p53, anti-p53 antibody; PT, prothrombin time; SUV, standardized uptake value on positron emission tomography scan; TACE, transarterial chemoembolization; TGF-β, transforming growth factor-β.

**Table 4:** Variables (*n*=6) that were most commonly found to be significant predictors of death in hepatocellular carcinoma in 72 studies

Variable	No. of studies in which variable was among the first five significant variables	No. of studies evaluating the variable	% of studies in which the variable was among the first five/total of studies
Portal vein thrombosis	22	32	69
Tumour size	20	33	61
AFP	20	41	49
Child–Pugh class	18	33	55
Bilirubin	15	24	63
CLIP score*	11	15	73

* Cancer of the Liver Italian Program (consists of portal vein thrombosis, tumour size, AFP and Child–Pugh class).

AFP, α-foetoprotein; CLIP, Cancer of the Liver Italian Program.

**Table 3:** A list of all variables (*n*=79) evaluated as predictors of death in 72 studies

Patient demographics (*n*=3)
*Age, Gender,* Ethnicity
Hepatic insufficiency (*n*=9)
*Child–Pugh class, MELD, albumin, bilirubin, PT, ICG clearance, LCSGJ liver damage scale*
Cirrhosis, hepatic encephalopathy
Portal hypertension (*n*=3)
*Ascites, varices*
Platelets
Tumour factors (*n*=28)
Portal vein thrombosis, metastases, lymph node involvement, gross HCC*, tumour size, tumour number, tumour histology, tumour stage, tumour echo†*, tumour margin*‡*, tumour doubling time, encapsulation*§*, SUV ratio, AFP, AFP-L3, DCP, LAK, NK, p53, IL-8, LINE-1, CRP, TGF-β, HGF, Milan criteria*¶
Tumour location, cholinesterase, PIVKA-II
Hepatocellular carcinoma staging classifications (*n*=10)
*CLIP score, LCSGJ stage, Okuda scale, BCLC scale, JIS scale, Modified JIS score, combination of staging systems*
GRETCH scale, CUPI scale, French score
Aetiological factors (*n*=6)
*Alcohol, hepatitis B, hepatitis C, viral load, HBeAg*
Genotype
Treatment (*n*=5)
*Untreated, treatment modality, surgery, TACE, delayed treatment*
Other (*n*=15)
*Performance status, symptoms, mode of detection, urea, creatinine, GGT, ALP, α-1 antitrypsin, centre of diagnosis, period of surveillance*
ALT, AST, haemoglobin, LDH, sodium

Variables in italics were among the first five significant variables on multivariable analysis in at least one study.

* Combination of number of tumours, size and extent of liver replacement.

† Echo pattern on ultrasound.

‡ Regular tumour margin on ultrasound.

§ Presence of capsule on ultrasound.

¶ Single tumour <5 cm or three tumours each <3 cm.

AFP, α-foetoprotein; AFP-L3, lens-culinaris agglutinin-reactive fraction of AFP; ALP, alkaline phosphatase; ALT, alanino aminotransferase; AST, aspartate aminotransferase, BCLC, Barcelona Clinic Liver Cancer; CLIP, Cancer of the Liver Italian Program; CRP, C reactive protein; CUPI, Chinese University Prognostic Index; DCP, des-gamma-carboxy prothrombin; GGT, ã-glutamyl transpeptidase; GRETCH, Groupe d'Etude et de Traitement du Carcinome Hepatocellulaire; HBeAg, hepatitis B envelope antigen; HCC, hepatocellular carcinoma; HGF, hepatocyte growth factor; ICG, indocyanine green clearance; IL, interleukin; JIS, Japanese Integrated System; LAK, lymphokine-activated killer activity; LCSGJ, Liver Cancer Study Group of Japan; LDH, lactate dehydrogenase; MELD, model for end-stage liver disease; NK, natural killer activity; p53, anti-p53 antibody; PIVKA-II, serum protein induced by vitamin K absence or antagonist II; PT, prothrombin time; SUV, standardized uptake value on positron emission tomography scan; TACE, transarterial chemoembolization; TGF-β, transforming growth factor-β.

When only the 15 ‘good quality’ studies are analysed (Table 6), the same most common predictors of death are observed as for the overall analysis, i.e. Child–Pugh class, AFP, portal vein thrombosis, CLIP score and tumour size. The most robust predictors were Child–Pugh class, AFP and portal vein thrombosis. As the majority of the studies (89%) evaluating Child–Pugh status divided patients by class (A, B, C) and not by score, the term Child–Pugh class is utilized in this review.

**Table 6:** Variables that were most commonly found to be significant predictors of death assessed in 15 ‘good’ quality studies*

Variable	No. of good studies in which variable was among the first significant ones	No. of good studies evaluating the variable	% of studies in which variable was among the first five/total studies
Child–Pugh class	8	11	73
AFP	8	10	80
Portal vein thrombosis	5	7	71
CLIP score	4	5	80
Tumour size	4	7	57

* Good quality studies included all the four major quality criteria [relevant baseline data shown, number of deaths reported, patients were included consecutively and ratio of number of deaths/number of variables >10 (i.e. no overfitting)].

AFP, α-foetoprotein; CLIP, Cancer of the Liver Italian Program.

When the 22 studies in which 100% of the patients had cirrhosis are analysed (11, 12, 14, 19, 23, 24, 26, 27, 32, 35, 36, 38, 47, 52, 53, 60, 62, 68, 69, 71, 78, 79) (Table 7), the most common predictors of death found in over three studies are the CLIP score, tumours that were untreated, tumour size, the Child–Pugh class, tumour number, AFP and portal vein thrombosis.

**Table 7:** Variables that were most commonly found to be significant predictors of death assessed in 22 studies in which 100% of the patients included had cirrhosis

Variable	No. of good studies in which variable was among the first significant ones	No. of good studies evaluating the variable	% of studies in which variable was among the first five/total studies
CLIP score	6	6	100
Tumour size	5	7	71
Child–Pugh class	5	11	45
Tumour number	4	6	67
AFP	4	10	40
Portal vein thrombosis	4	10	40

AFP, α-foetoprotein; CLIP, Cancer of the Liver Italian Program.

The number of studies that included mostly compensated or decompensated cirrhotic patients was small. However, as shown in Table 8, in both groups of patients, the predictors of death included both liver-related and tumour-related factors. When patients were separated by advanced or non-advanced tumour status, the most important predictors of death in patients with advanced tumours were portal vein thrombosis, AFP, bilirubin and lack of treatment (Table 9). The number of studies analysing significant predictors of death in patients with non-advanced tumours was small.

**Table 9:** Variables significant in studies including mostly advanced tumours or mostly non-advanced tumours

Advanced tumours (15 studies)				Non-advanced tumours (7 studies)			
Variable	No. of significant studies	No. of studies evaluated	%	Variable	No. of significant studies	No. of studies evaluated	%
Portal vein thrombosis	6	9	67	Albumin	2	2	100
AFP	5	8	63	Tumour size	2	2	100
Bilirubin	4	6	67	DCP	2	2	100
Untreated	4	6	67	Age	2	3	67

AFP, α-foetoprotein; CLIP, Cancer of the Liver Italian Program; DCP, des-gamma-carboxy prothrombin.

**Table 8:** Variables significant in studies including mostly compensated or mostly decompensated patients

Compensated cirrhosis (13 studies)				Decompensated cirrhosis (5 studies)			
Variable	No. of significant studies	No. of studies evaluated	%	Variable	No. of significant studies	No. of studies evaluated	%
DCP	4	5	80	Tumour size	2	2	100
Bilirubin	4	5	80	AFP	2	2	100
Child–Pugh class	4	7	57	Albumin	2	3	67
AFP	4	11	36				
CLIP	3	4	75				
Treatment received	3	4	75				
Tumour size	3	4	75				

AFP, α-foetoprotein; CLIP, Cancer of the Liver Italian Program; DCP, des-gamma-carboxy prothrombin.

## Discussion

Hepatocellular carcinoma can arise in both non-cirrhotic and cirrhotic livers. In the Orient, where hepatitis B or toxins are the most common underlying causes, HCC commonly arises in the absence of cirrhosis (80, 81). In the west, where hepatitis C and alcohol are the most common underlying liver diseases, HCC arises mostly in the setting of cirrhosis (81–83). Prognosis is predictably worse in patients with underlying cirrhosis (81, 84). Even in patients with cirrhosis (without HCC), it has recently been shown that the prognostic factors, survival and causes of death differ significantly between those with compensated and decompensated cirrhosis (2) and that these two entities should be considered separately both in clinical practice and in clinical research (85). In fact, HCC is an independent predictor of death in patients with decompensated cirrhosis (2).

Prognosis is an essential part of the assessment of patients with HCC. It allows the patient to make important decisions, both therapeutic and other and it allows for risk stratification such that different therapies can be investigated according to risk. However, prognostic studies in HCC are often unsatisfactory because patients included are heterogeneous, particularly with regard to the presence (or absence) of underlying cirrhosis, and the results are therefore not widely applicable. Because most of the cases of HCC in the USA occur in the setting of cirrhosis, it is important to determine the prognostic variables in this subset of patients with HCC. Unfortunately, many of the prognostic studies in HCC include patients with and without cirrhosis. In fact, of the 159 articles retrieved for this analysis, at least 45 (28%) were excluded because more than 20% of the patients did not have cirrhosis or the presence (or absence) of cirrhosis was not mentioned. Even within the 72 studies selected for analysis, only 22 (31%) of them clearly stated that they included only patients with cirrhosis.

One of the strengths of this systematic review is that it analysed predictors of death in HCC, specifically in studies in which ≥80% of the patients had underlying cirrhosis. Furthermore, it aimed to analyse whether the predictors differed in patients with compensated vs. decompensated cirrhosis.

Despite methodological problems in the evaluated studies (none met all quality criteria and only 21% of them met important quality criteria), this systematic review allowed for the identification of the ‘robust’ predictors of death. As could have probably been predicted, these were both tumour related (portal vein thrombosis, tumour size and AFP) and cirrhosis related (mainly, the Child–Pugh class). The strength or the robustness of a predictor is given by the ratio between the number of studies in which each variable was significant and the number of studies in which it was assessed. With a larger number of studies, a large ratio is an indirect measure of the validity as each study that confirms the predictive value of a variable provides indirect proof of its validity (2). This robustness, which is independent of the quality of the studies, was particularly true for portal vein thrombosis, tumour size and Child–Pugh class, each of which was evaluated in more than 30 studies with a ratio >50%, i.e. more than half of the studies proved them to be among the first five most significant variables on a multivariable analysis. Importantly, the same parameters were the most frequent significant variables when only the studies in which 100% of the patients included had cirrhosis and when ‘good’ quality studies were analysed. In the analysis of good quality studies, one of the most robust predictors of death was the AFP.

Interestingly, one of the most commonly used HCC staging systems, the CLIP system (4), includes all four of these predictors (portal vein thrombosis, tumour size, Child–Pugh class and AFP) and, in fact, the CLIP staging system itself was identified as the sixth most common predictor of mortality, being among the first five significant variables in 11/15 studies (73%). Six of the 11 studies identifying the CLIP score as an independent predictor were of Italian origin (24, 26, 36, 47, 53, 72). Another commonly used HCC staging system, and the one recommended in recently published guidelines (81), is the Barcelona Clinic Liver Cancer (BCLC) system (5), which was only evaluated in three studies, but was nevertheless found to be among the first five significant variables in two (67%) of them. This staging system also includes three of the predictors (tumour size, Child–Pugh class and portal vein thrombosis). Although it does not include AFP, the BCLC staging system has the advantage of including other parameters such as performance status (found to be an important predictor in 6/8 or 75% of the studies in which it was assessed) and, importantly, of tailoring other prognostic factors to different tumour stages (early vs. intermediate/advanced) and to Child–Pugh class. For example, portal pressure, as determined by the hepatic venous pressure gradient, which was not directly evaluated in any of the studies in this analysis, was shown to be an independent predictor of death in early stages (patients subjected to resection) (86, 87) and is included in the BCLC system but only in Child A patients with resectable tumours (5). In the BCLC multivariable analysis study that was performed to identify the predictors of death in intermediate/advanced stages, an AFP of >35 ng/ml was not significant on univariate analysis (23), whereas in the CLIP staging system an AFP of >400 ng/dl had an independent prognostic value (4). As has been recently discussed, the prognostic utility of AFP is unclear in part due to the use of heterogeneous cut-off levels and also due to its variable sensitivity (88, 89). For example, in a recent study of 1158 patients (60% Child–Pugh A), even though the specificity of an AFP level >600 ng/ml in predicting survival was 93%, its sensitivity was only 23% (89).

Therefore, although the BCLC model has been reported to be superior to alternate models for the prediction of survival in HCC, including the Okuda, CLIP, tumour node metastasis, Japanese Integrated System, Groupe d'Etude de Traitement du Carcinoma Hepatocellulaire and Chinese University Prognostic Index models (61, 67), three of which include the AFP, it would be interesting to see whether the AFP (as a continuous variable) adds to the prognostic value of the BCLC. The appropriate cut-off level and the group of patients in which the AFP may be helpful remains to be determined, although our analysis would suggest that it appears to be more useful in advanced tumour stages (Table 9).

The importance of determining prognosis at different stages of cirrhosis cannot be overemphasized (2). We had hypothesized that in the compensated patients factors related to the tumour would be more important, whereas in the decompensated patient, both liver- and tumour-related factors would be important. However, in a subanalysis of studies including mostly patients with compensated or decompensated cirrhosis (Table 8), both tumour-related and cirrhosis-related predictors appeared to be predictive of death in both groups. However, the number of studies is too small to draw firm conclusions, particularly regarding patients with mostly decompensated cirrhosis and those with mostly non-advanced tumour stage.

Our study is limited by the design and prognostic parameters chosen in each of the studies included in this analysis as well as the heterogeneous group of patients included with both treated and untreated tumours. Furthermore, although the choice of the 66% cut-off for the definition of ‘mostly decompensated cirrhosis’ or ‘mostly non-advanced tumour’ was in part arbitrary, it can be justified because even at this cut-off there were too few studies available in the >66% non-advanced tumour or >66% decompensated cirrhosis categories to obtain definitive results.

Future studies on the prognostic indicators of HCC should include patients either with or without cirrhosis. Studies that will apply to patients with HCC seen in western countries should include only patients with cirrhosis and the prognostic variables should be assessed separately for the different stages of cirrhosis, at a minimum, separating those with compensated and those with decompensated cirrhosis. The study quality should be optimized by incorporating most, if not all, of the criteria listed in Table 1. Additionally, parameters identified by the majority of the studies included in this analysis should be included in such studies.

## Abbreviations

AFP: α-foetoprotein
BCLC: Barcelona Clinic Liver Cancer
CLIP: Cancer of the Liver Italian Program
HCC: hepatocellular carcinoma.
